# Fibrous and membranous tissues of the tarsal tunnel; quantitative 3D anatomy using a digital microscribe

**DOI:** 10.1007/s00276-026-03891-1

**Published:** 2026-05-26

**Authors:** Georga K. Bruechert, Casper G. Thorpe Lowis, William H. B. Edwards, Quentin A. Fogg

**Affiliations:** 1 The University of Melbourne, Melbourne, VIC Australia; 2 The Epworth Hospital, Richmond, Melbourne, VIC Australia

**Keywords:** Tarsal tunnel, Septae, Fibrous, Tendon sheath, Tarsal tunnel syndrome

## Abstract

**Purpose:**

The aetiology of tarsal tunnel syndrome is not always clear. Treatment commonly involves a single release of the flexor retinaculum, but post-operative outcomes are not always successful. This may be due to the lack of understanding the tissues that may compress the neurovasculature. The aim of this study was to define the tissues that separate the contents of the tarsal tunnel.

**Methods:**

Feet from embalmed body donors (*n* = 15; mean age = 83.9 ± 10.2 years; female = 9, male = 6) were examined. One underwent magnetic resonance imaging. All were dissected and modelled in virtual three-dimensional space.

**Results:**

The tarsal tunnel was divided into at least five spaces by a fibrous skeleton, formed by septae that were much thicker than previously understood.

**Conclusion:**

These data strongly suggest that the fibrous skeleton may compress the neurovasculature and may need to be considered in the diagnosis and management of tarsal tunnel syndrome. Surgically, this may more precisely inform which tissues need to be targeted for tarsal tunnel release.

## Introduction

**Fig. 1:**
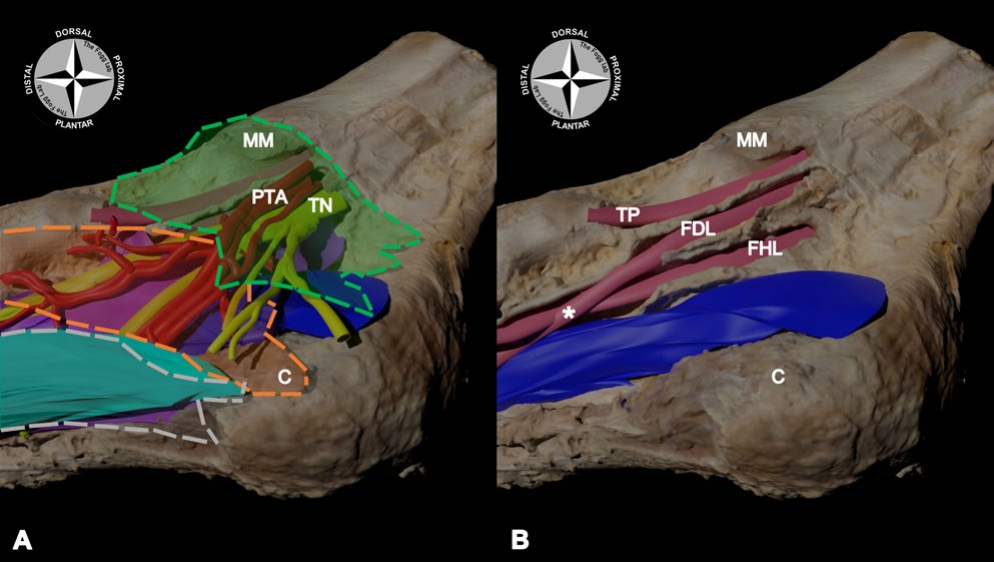
Digital model of the tarsal tunnel from a medial-plantar view.** A** – Within the literature the flexor retinaculum (transparent green with dotted border) is described as forming the medial roof of the tarsal tunnel. It is typically the only tissue to be released during the treatment of tarsal tunnel syndrome to relieve pressure on the underlying neurovasculature coursing through the tunnel. These include the posterior tibial artery and its venae comitantes (PTA; red), the tibial nerve (TN; yellow), and their associated branches.** B** –Lateral to these structures, the tendons of the tibialis posterior (TP), flexor digitorum longus (FDL), and the flexor hallucis longus (FHL) also course through the tarsal tunnel. Distally along their course, the FDL and FHL cross at the level of the chiasma plantare (white star). Transparent orange with dotted border: abductor hallucis muscle; transparent grey with dotted border: plantar aponeurosis; light blue: flexor digitorum brevis muscle; dark blue: quadratus plantae muscle; MM: medial malleolus; C: calcaneus

Tarsal tunnel syndrome (TTS) is a debilitating condition with various symptoms, including paraesthesia, pain, cramping, or anaesthesia [1, 2]. The aetiology of TTS is not always clear, and there is currently no gold standard technique for diagnosis and treatment. In the reviewed literature, the most common treatment was the release of the flexor retinaculum [1–3], the fibrous tissue forming the medial roof of the tarsal tunnel [4–7] (Fig. 1A). This procedure was not always successful, with reports of 42–92% of patients having *some* improvements [3, 8, 9], yet only 50% of patients reporting no symptoms post-operatively [9]. As there are no consistently used assessment tools, it is not clear whether the outcomes of these cases were fully satisfactory. Were patients able to return to completely normal function, or adequate function with pain, or continued dysfunction and pain that was not reported?

The tarsal tunnel is a complex osteo-fibrous space that contains neurovasculature – the posterior tibial artery (PTA) and its associated veins (venae comitantes), and the tibial nerve (TN) and their branches – and the tendons of the tibialis posterior (TP), flexor digitorum longus (FDL) and flexor hallucis longus (FHL) muscles [4, 5, 10] (Fig. 1).

Within the tarsal tunnel, it is not clear how the tendons are separated from one another or the neurovasculature. Most commonly, the tendons have been suggested to be separated from one another only by tendinous sheaths lateral to the flexor retinaculum [5, 11–14]. These fibrous tissues have formerly been identified using variable techniques, including dissection, arthroscopic surgery, and medical imaging [4, 6, 7, 10–13, 15]. The reviewed studies were typically limited to one modality, and commonly focused on only one or few structures, not the entirety of the tarsal tunnel [4, 6, 7, 10, 13, 15, 16]. These studies investigated the bifurcation patterns of individual nerves and/or arteries, or only considered individual structures that may impact them. Further study is required for a more detailed understanding of all structures that may impact the neurovasculature.

Anatomical studies of various body regions have been enhanced in recent years by the incorporation of digital microscribe modelling. A microscribe allows directly observed structures to be modelled in a virtual three-dimensional (3D) space. Accurate reconstruction of non-geometric structures with irregular contours, such as muscles and ligaments, has been clearly demonstrated through the use of a digital microscribe [17, 18]. To date, this technique has not been utilised to reconstruct and assess the fibrous and irregular tissues of the tarsal tunnel.

Therefore, the aim of this study was to analyse the tissues separating the tendons and neurovasculature within the tarsal tunnel, using a novel approach with dissection and 3D modelling. Obtaining a clearer understanding of the tissues that separate the contents of the tarsal tunnel may lead to identifying structures that could contribute to the development of TTS.

## Materials and methods

Feet from body donors (mean age = 83.9 ± 10.2 years; female = 9; male = 6) that had been fully embalmed (Anatomical Series, Genelyn, Australia) by the University of Melbourne Body Donor Program were examined in accordance with *Part IV of the Human Tissue Act* (1982) and with local ethics approval (project ID: 12292). A fascicular dissection strategy was utilised to assess the fibrous tissue that separated the contents of the tarsal tunnel. This allowed for two-dimensional (2D) surface models (non-volumetric) of these 3D structures to be reconstructed within a 3D virtual space.

Each specimen was pre-screened by CT imaging. Those with abnormalities that would influence this project (previous surgery, tarsal tunnel or plantar foot deformation) were excluded (*n* = 0). The DICOM data were segmented to allow the production of 3D skeletal models, used as a base for visualisation of soft tissue models.

Using standard surgical instruments, all medial-proximal skin and subcutaneous tissue was removed at the level of the tarsal tunnel. The flexor retinaculum was identified according to the direction of fibres coursing from the medial malleolus to the calcaneus. Further dissection of the plantar fibres of the flexor retinaculum was carried out in the plantar direction. This exposed the more lateral structures of the tarsal tunnel, the abductor hallucis (AbH) muscle and the neurovasculature. These structures were reflected from the proximal plantar foot. Tissues located further distally along the plantar foot that possessed a clear fascicular structure were retained.

Longitudinal incisions were made along the flexor retinaculum at the level of the TP and FDL tendinous sheaths. The incisions were made at what was identified as the proximal border of the retinaculum and were extended along the dorsal and plantar walls of each of the tendinous sheaths. The TP and FDL tendons were excised at the level of the tunnel, exposing the lateral surfaces of their tendinous sheaths.

The fibrous tissue medial to the FHL tendon, forming the medial wall of its tendinous sheath, was incised and excised, exposing the FHL tendon. This tendon, as well as the soft tissue surrounding it, was completely removed up to the level at which the FHL and FDL tendons cross at the chiasma plantare (also known as the Knot of Henry) (Fig. 1B). This provided direct visualisation of the dorsal, plantar, and lateral walls of the FHL tendinous sheath.

As the tarsal tunnel was dissected, each structure of interest was modelled within a virtual 3D environment, using a MicroScribe G2X Digitiser (Immersion Corp., USA) and Rhinoceros 4.0 Software (Robert McNeel and Associates, USA). A series of coordinate points were digitised along the surface of each structure, ensuring that a coordinate point was recorded when the nature of the structure changed (e.g., when the surface began to change planes). The coordinate points formed a line when joined together. Multiple lines were reconstructed along a 3D surface to account for any contours and for the irregular nature of such 3D structures. Multiple lines were lofted together to reconstruct a 2D representation of the surface of the 3D structure. This technique provided a virtual 3D representation of all structures of interest, which could be rotated and manipulated within a virtual 3D environment.

Two additional coordinate points were modelled from pins that had been inserted into the most medial point of the medial malleolus (Pin A) and the medial tuberosity of the calcaneus (Pin B). These were used as reference points so that the virtual 3D reconstructions of each tissue could be realigned in the instance the specimens had been moved in between microscribing and dissecting.

The thickness of all fibrous and membranous tissues of interest was measured using a digital calliper (Clarke International, UK). Measurements were taken at locations where the observer had noted that the thickness had appeared to change. Multiple measurements of each structure were obtained and the mean values from each round of measurement were compared by carrying out a one-way ANOVA. The average thickness of each tissue was compared with a one-way ANOVA to determine which were thicker, and if the thickness changed across their course. Statistical analysis was carried out with a significance of 0.05. Measurements were reported as averages (+/- standard deviation) when calculated from more than one specimen.

A single specimen was prepared for magnetic resonance (MR) imaging, with the addition of a contrast gadopentetate dimeglumine (Magnevist, 10mM) medium, injected into the sheaths of the TP, FDL and FHL tendons. Muscles that course through the tunnel were identified proximal to the tip of the medial malleolus (~ 20 mm), at which point their sheaths were opened allowing direct visualisation for insertion of a needle. The needle was sutured in place to ensure a seal was created. For each sheath, 2.5-5.0mL of Magnevist was injected using a syringe and needle. A 7T MR image (SIEMENS MRI7T-2008) was generated at The Melbourne Brain Centre Imaging Unit.

## Results

**Fig. 2:**
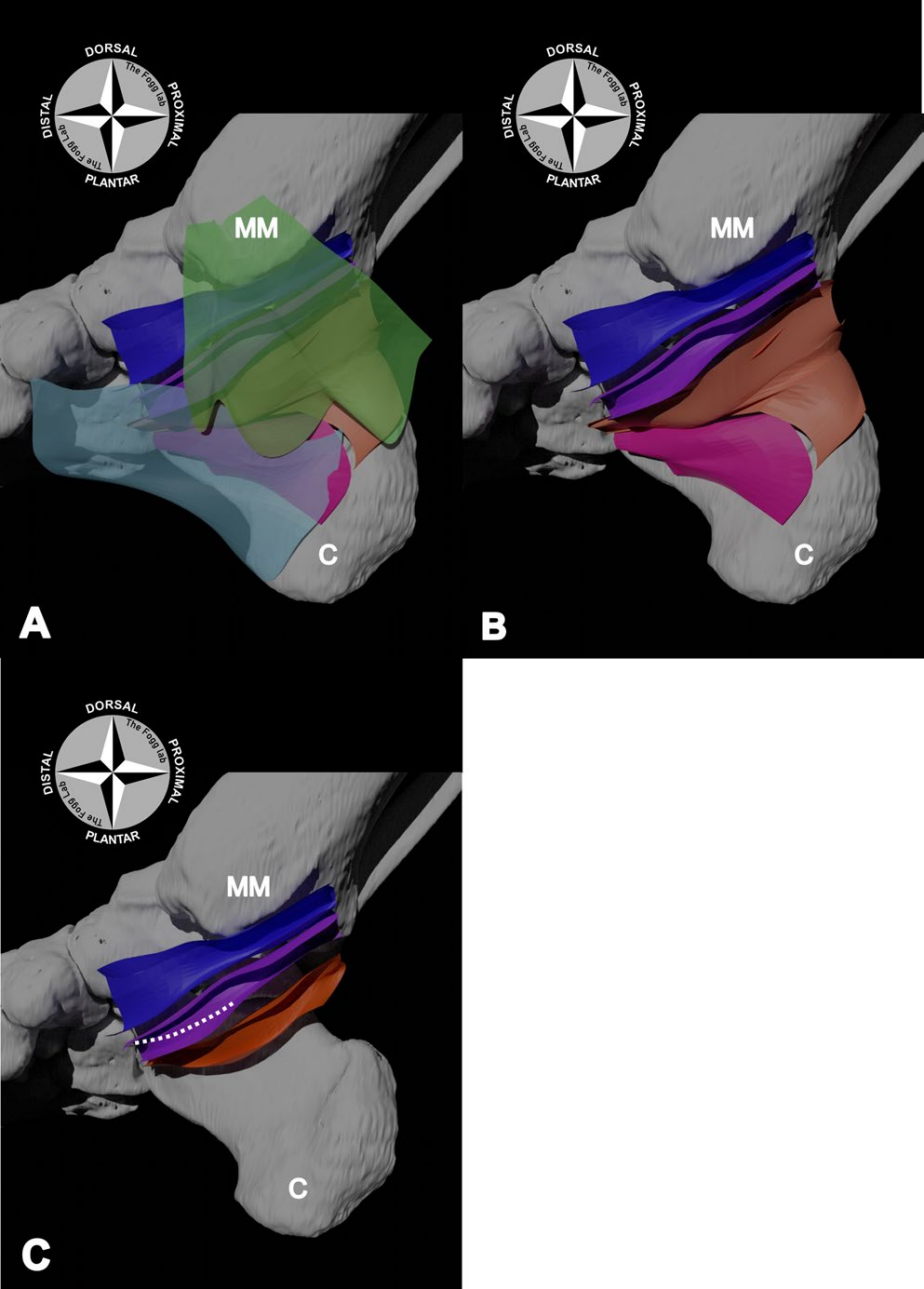
Digital model of the boundaries of the tarsal tunnel and tendinous sheaths.** A** – The flexor retinaculum (transparent green) and the abductor hallucis muscle (transparent light blue) formed the medial roof of the tarsal tunnel, as well as the medial walls of the tibialis posterior (dark blue) and flexor digitorum longus (FDL) tendinous sheaths (purple).** B** – Plantar to the FDL sheath, a broad band of tissue formed the medial roof of the flexor hallucis longus (FHL) tendon sheath (orange). This tissue had a close relationship with the quadratus plantae muscle (pink), which attached to the calcaneus along its plantar border.** C** – Most lateral and plantar to the sustentaculum tali (dotted white line: plantar border), the remaining walls of the FHL tendon sheath were identified (orange). MM: medial malleolus; C: calcaneus

**Fig. 3:**
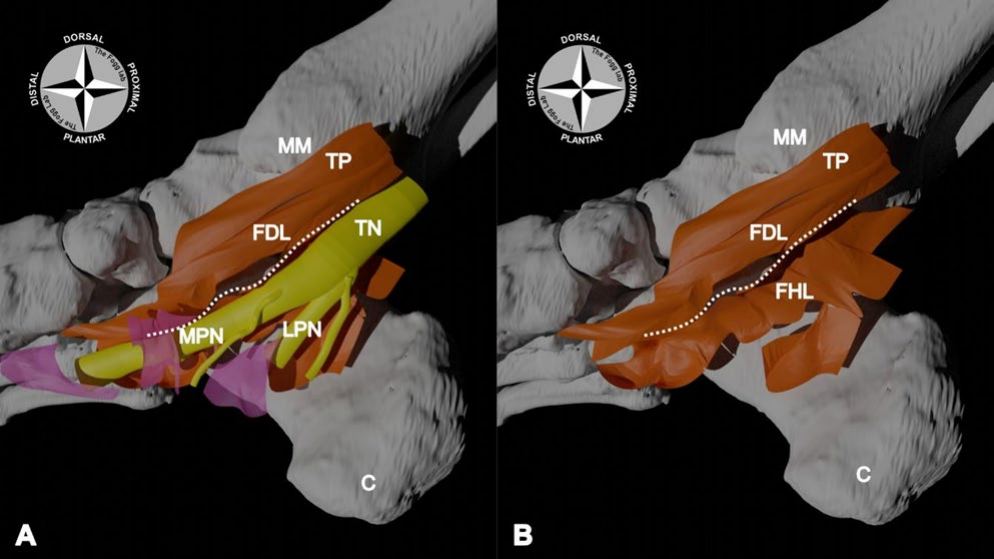
Digital model of the medial walls of the relationship between the nerves and the tendinous sheaths.** A** – Upon the removal of the posterior tibial artery and its branches (not pictured), the tibial nerve (TN) and medial plantar nerve (MPN) coursed along the plantar side of the plantar septum of the flexor digitorum longus (FDL) tendon sheath (dotted white line). The lateral plantar nerve (LPN) branched off the TN. Both the MPN and LPN continued distally into the plantar foot, deep to other fascial tissues (transparent pink).** B** – Directly deep/lateral to the TN and its branches was the medial wall of the flexor hallucis longus (FHL) tendon sheath. The plantar septum of the FDL tendon sheath (white dotted line) separates these two tendon sheaths. MM: medial malleolus; C: calcaneus; TP: tibialis posterior tendon sheath

**Fig. 4:**
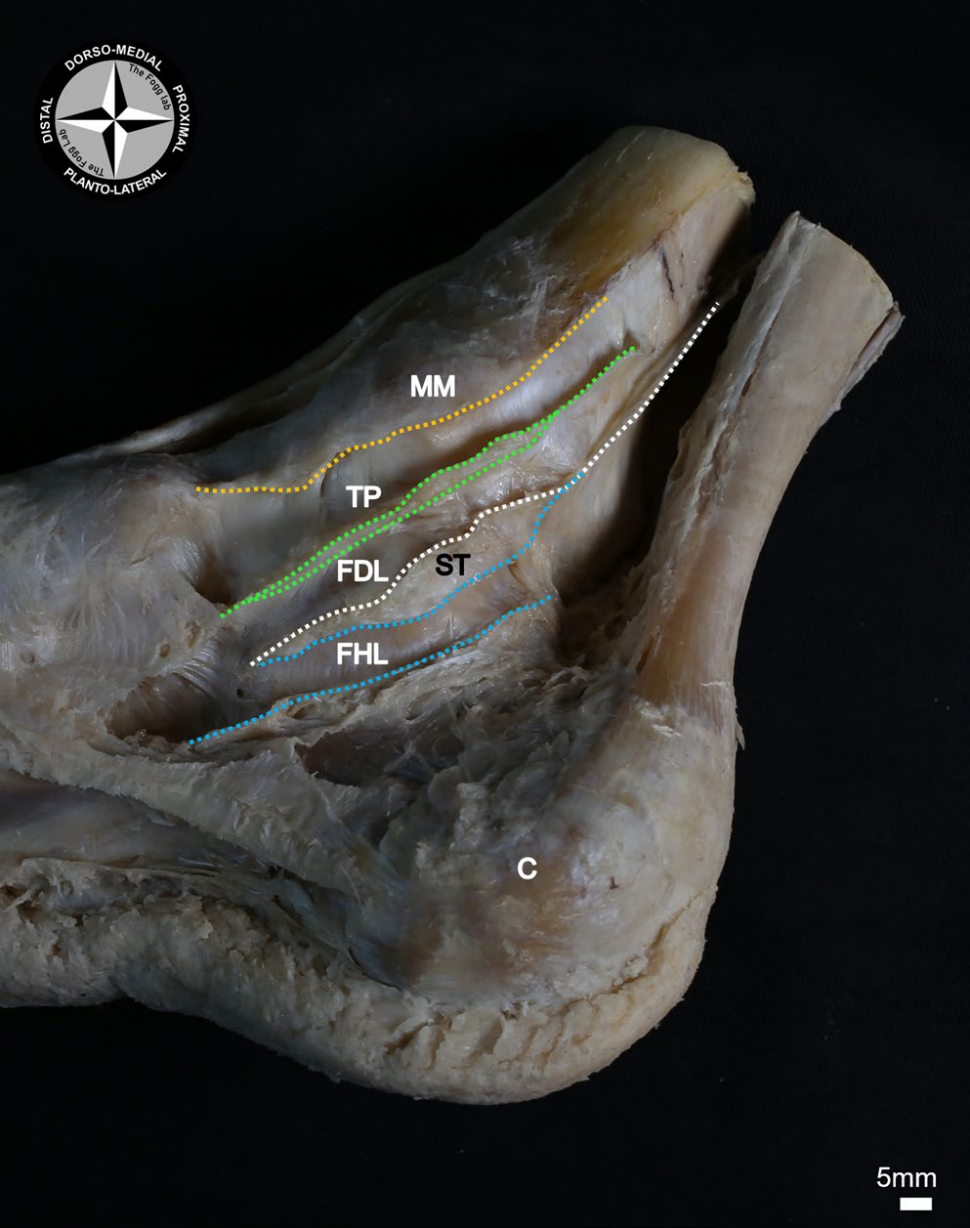
A medial-plantar view of the septae of the tendinous sheaths. The tibialis posterior (TP) tendon was encapsulated within its own tendinous sheath, running along the medial-plantar side of the medial malleolus (dotted orange line), and separated from the flexor digitorum longus (FDL) tendon by a septum (dotted green lines). Plantarly to the FDL tendon another septum (dotted white line) coursed along the medial-dorsal border of the sustentaculum tali (ST). Planto-lateral to the ST was the tendinous sheath of the flexor hallucis longus (FHL) tendon, with the ST forming its more dorsal septum (dorsal dotted blue line). The plantar FDL septum discontinued at the distal end of the ST, indicating the point at which the two tendinous sheaths of the FDL and FHL tendons became one. The FHL tendinous sheath was enclosed by a plantar septum that ran along the medial side of the calcaneus (plantar dotted blue line). MM: medial malleolus; C: calcaneus

The TP tendon was excise from its attachment to the navicular tuberosity, whilst leaving its tendinous sheath intact. The dorsal wall of the sheath was formed by the bony prominence of the medial malleolus, whilst the lateral wall was formed by the medial malleolus, talus and the navicular bone. The plantar wall of the sheath was formed by a thick fibrous septum (*n* = 15; 100%; mean thickness = 1.47 ± 0.83 mm). This septum coursed medially from the flexor retinaculum to attach to the medial surfaces of the distal tibia and calcaneus, separating the TP tendon from the more plantar FDL tendon (Figs. 2, 3 and 4).

The FDL muscular fibres merged into a distal tendon at the proximal border of the flexor retinaculum, and therefore the tarsal tunnel. The FDL tendon was excised from this level to the chiasma plantare. The distal border of the tarsal tunnel was therefore further defined, as this was the point where the lateral wall of the FDL tendinous sheath ended, and where the more lateral FHL tendon was now visible at the chiasma plantare.

Upon the excision of the FDL tendon, the remaining walls of its tendinous sheath were directly visible. Its dorsal wall was formed by the fibrous septum previously described to separate the FDL and TP tendons.

The plantar wall of the FDL tendon sheath was formed by a thick fibrous septum (*n* = 15; 100%; mean thickness = 0.9 ± 0.59 mm) that, like the dorsal septum, coursed from the flexor retinaculum medially, to the bony structures forming the lateral wall (the talus, the sustentaculum tali of the calcaneus, and the proximal end of the navicular bone; Figs. 2, 3 and 4). Upon the removal of the posterior tibial artery (PTA) and its branches, the tibial nerve (TN) and/or medial plantar nerve (MPN) were identified to course directly plantarly alongside the plantar FDL septum (Fig. 3).

Upon reflection of the neurovasculature, a thick fibrous tissue (*n* = 15; 100%; mean thickness = 0.39 ± 0.18 mm) was identified. It was positioned between the neurovasculature and FHL tendon, forming the medial wall of the FHL tendinous sheath (Figs. 2 and 3). The more plantar fibres of this medial wall were covered medially by the quadratus plantae (QP) muscle. Upon excision of this muscle, it was seen to have overlain/terminated at the border of a thick plantar septum (*n* = 15; 100%; mean thickness = 0.72 ± 0.46 mm) that formed the plantar wall of the sheath.

Both the lateral and dorsal walls of the FHL tendinous sheath were formed by the medial body of calcaneus and the sustentaculum tali of the calcaneus, respectively. The FHL tendinous sheath was positioned plantar-lateral to the FDL tendon and its sheath (Figs. 2 and 4).

**Fig. 5:**
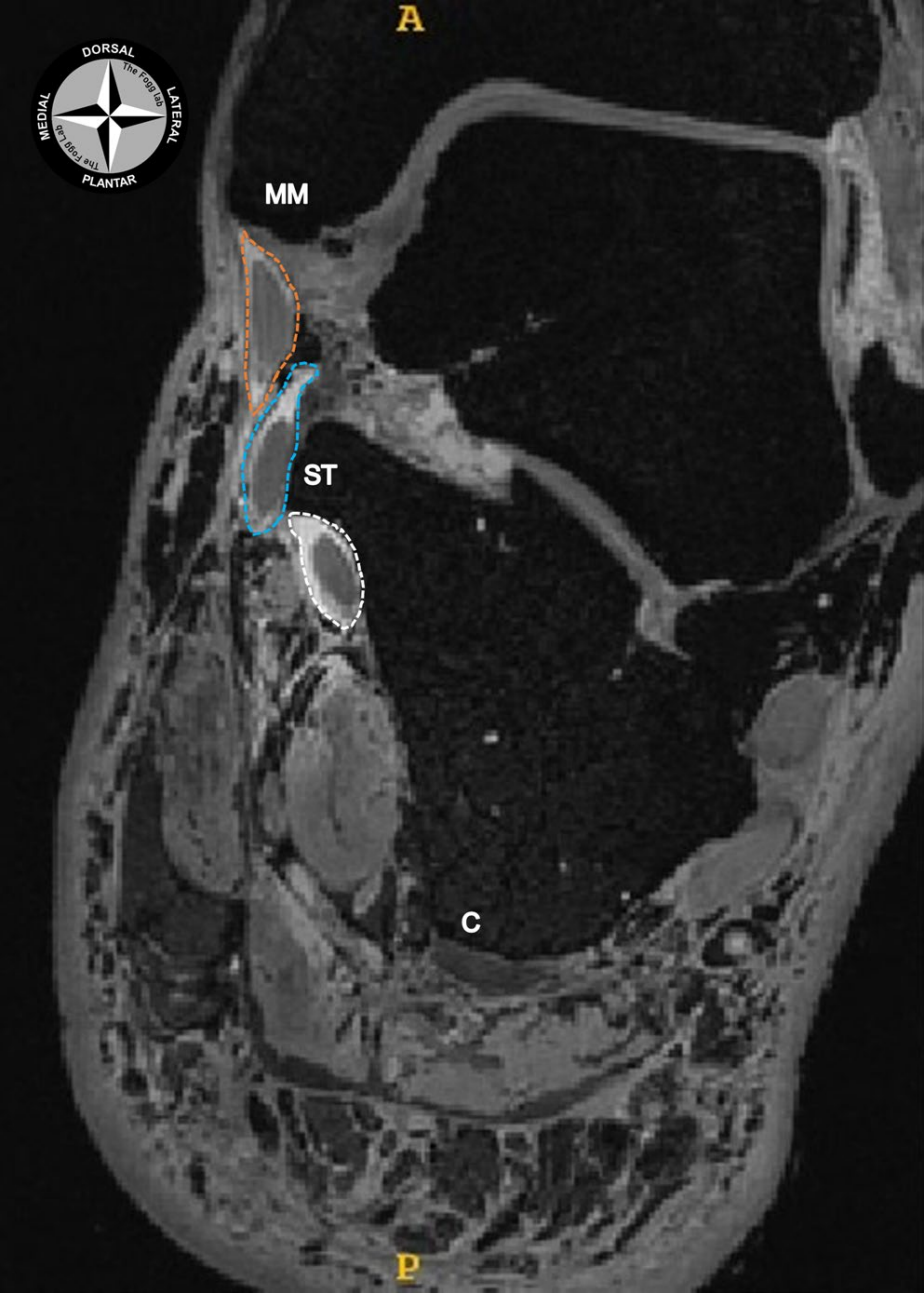
A coronal section of an MR image at the level of the tarsal tunnel. The tendinous sheaths of the tibialis posterior (orange dotted line), flexor digitorum longus (blue dotted circle) and flexor hallucis longus (white dotted line) tendons were injected with Magnevist. This enhanced the visualisation of the separate tendinous sheaths at the level of the sustentaculum tali (ST) within the tarsal tunnel. MM: medial malleolus; C: calcaneus

**Fig. 6:**
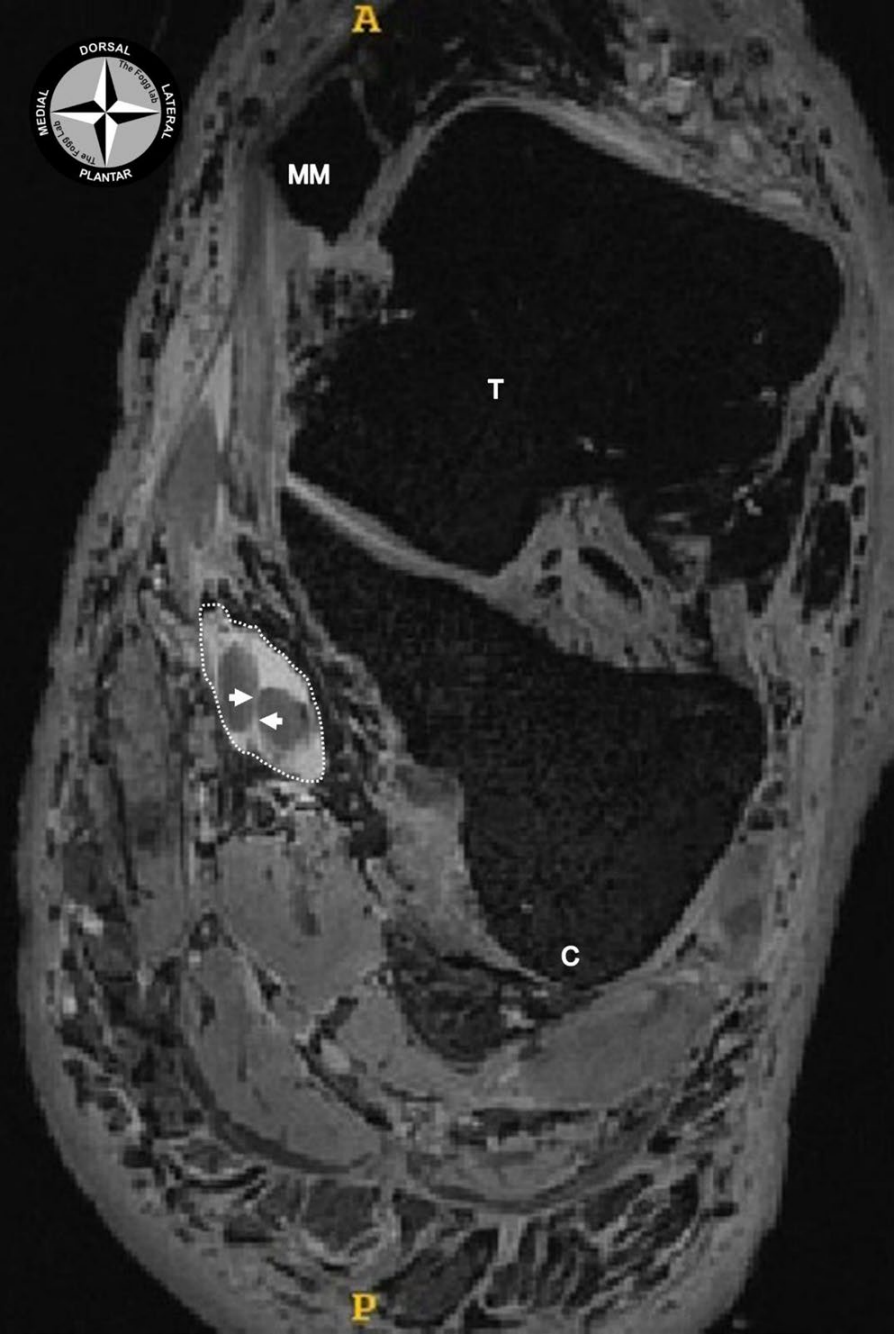
A coronal section of an MR image at the level of the chiasma plantare, with the tendons of the flexor digitorum and hallucis longus tendons crossing over. The tendinous sheaths of these tendons were injected with Magnevist to enhance the visualisation of their spatial relationship. At this level only a thin membranous tissue (white arrows) was separating the two tendons as they coursed in their common tendinous sheath (white dotted circle). MM: medial malleolus; C: calcaneus; T: Talus

Distal to the tarsal tunnel, both the FHL and FDL tendinous sheaths discontinued (*n* = 15; 100%), allowing the tendons to cross over at the level of the chiasma plantare. At this level, these two tendons were separated from one another only by a thin membranous tissue in most cases (*n* = 14; 93%; mean thickness = 0.10 ± 0.07 mm). This tissue was not modelled as it was too thin and displaced upon the removal of the tendons, but it was visible within the MR images (Figs. 5 and 6).

Fibrous and membranous tissues were measured multiple times. When assessed collectively, there was no statistically significant difference (*p* > 0.05) between rounds of measurement for any assessed structure, suggesting that, on an intra-observer basis, the results were accurate and reproducible.

The septum between the TP and FDL tendons was significantly (*p* < 0.05) thicker than all of the other fibrous and membranous tissues measured in the current study (Table 1). The plantar septae of the FDL and FHL tendon sheaths were statistically similar (*p* > 0.05) in thickness, but they were both significantly thicker than the medial wall of the FHL tendon sheath (*p* < 0.05). The membrane between the FDL and FHL tendons distal to the level of the tarsal tunnel was significantly thinner (*p* < 0.05) than all fibrous tissues measured in the current study, except for the medial wall of the FHL tendon sheath, to which it was of a statistically similar thickness (*p* > 0.05).

**Table 1:** Mean measurements (± SD) of the fibrous septae contributing to the tendinous sheaths of the tarsal tunnel, and the membranous tissue separating the long flexor tendons at the level of the chiasma plantare (CP)

	Thickness					
	Proximal	First third	Middle	Second third	Distal	Group
Dorsal FDLts septum	1.07 ± 0.44	1.33 ± 0.65	1.15 ± 0.43	1.44 ± 0.68	2.27 ± 1.11	A
Plantar FDLts septum	0.83 ± 0.65	0.95 ± 0.53	1.23 ± 0.57	1.07 ± 0.64	0.52 ± 0.38	B
Medial FHLts septum	0.31 ± 0.19	–	0.37 ± 0.19	–	0.41 ± 0.18	B
Plantar FHLts septum	0.70 ± 0.56	0.69 ± 0.39	0.72 ± 0.23	0.92 ± 0.60	0.60 ± 0.38	C
Membrane between FDL and FHL tendons at CP	0.10 ± 0.07					C

Measurements were consistently taken at the proximal and distal margins of the septae, as well as at the midline. For all septae, except the medial septum of the flexor hallucis longus tendon sheath (FHLts), measurements were also taken at the first and second third along their course. FDLts: flexor digitorum longus tendon sheath. Means that do not share a group letter are significantly different in thickness (*p* < 0.05), whilst those that have the same letter, are statistically similar (*p* > 0.05)

## Discussion

 The neurovasculature of the tarsal tunnel was more closely associated with surrounding fibrous and muscular tissues within the tunnel than previously thought. The current data were used to create a 3D model which could be used to describe the fibrous tissues forming the walls of the TP, FDL and FHL tendinous sheaths, and the proximal fibres of the AbH and QP muscles. Of particular importance were the tendinous sheaths, which were thicker than previously appreciated, hence reducing the available space within the tunnel. The measurement techniques used throughout the study were consistently accurate, as supported by the statistical analyses of multiple rounds of measurement, suggesting that these techniques should be utilised in future studies, facilitating more realistic comparison between studies. A paucity of such studies in the reviewed literature made comparison of the current data extremely limited. Being able to visualise the thickness of the septae and tendinous sheaths may be beneficial in being able to determine whether they are causing compression of surrounding neurovascular structures. This may be addressed in future studies using ultrasound or MR imaging.

The neurovasculature of the tarsal tunnel, particularly the TN and its branches, had a close relationship with a thick fibrous septum dorsally, and a thick lateral fibrous tissue. Although one previous study suggested that the neurovasculature ‘attached’ to septae that come off the flexor retinaculum and attached to the calcaneus [19], the role of these septae in separating the neurovasculature was not addressed. In the present study, the dorsal septum separated the neurovasculature from the FDL tendon, contributing to its tendinous sheath. This tendinous sheath consisted of another septum identified more dorsally, separating the FDL and TP tendons. Although tendinous sheaths have previously been suggested to encase the FDL and TP tendons [12–14, 20–23] within the tarsal tunnel [5, 11, 14, 21], most earlier studies [23, 24] and anatomical textbooks [5, 25] did not clearly identify or describe the tissues that compose these walls.

The septae composing the dorsal [26, 27] and plantar walls [26] of the FDL tendinous sheath have been mentioned in the reviewed literature, though not to the same detail or context as the present study. The TP and FDL tendinous sheaths were more frequently described as separate from one another and the surrounding tissues, including the flexor retinaculum [11, 13, 14, 21, 22, 26]. This is not consistent with the present findings, where the dorsal and plantar septae surrounding the FDL tendon continued from the flexor retinaculum, which itself formed the medial wall of both the FDL and TP tendinous sheaths. This was subtly suggested in one previous study, where several septae were reported to continue lateral to the flexor retinaculum to attach to the medial calcaneus [19].

Similarly, the tissues that make up the walls of the more lateral FHL tendinous sheath have not previously been described clearly, including the fibrous tissue forming its medial wall and the septum forming its plantar wall [28, 29]. The septum between the FDL and FHL tendons abruptly ended distal to the tarsal tunnel. These tendons were then only separated from one another by a thin membranous tissue, as they began to cross at the chiasma plantare. This finding was as expected, as it is well established in the literature that at the chiasma plantare, the FHL and FDL tendons form a chiasm and can sometimes interdigitate [22, 28, 30]. The fibrous tissue forming the medial wall of the FHL tendon sheath was of particular interest as it had no contribution from the flexor retinaculum, but rather a close relationship with the neurovasculature. It was also thick and broad, suggesting a clear mechanical influence over the movement of adjacent structures.

All identified fibrous tissues contributing to the walls of tendinous sheaths were much thicker than expected. Typically, earlier studies only identified the tendinous sheaths, but then directly incised them without further investigation [13, 14, 20, 22, 23]. Therefore, the findings of the current study could contribute to a better understanding of the potential involvement of such tissues in compressing neurovascular structures. At the least, an impression of more “free space” within the tunnel should be reduced by consideration of the space occupied by the thicker-than-expected fibrous tissues.

The QP muscle is generally understood to be within the central aspect of the plantar foot [5]. Therefore, the identification of the QP muscle within the tarsal tunnel, and its close spatial relationship with the FHL tendinous sheath, was not expected. Upon further investigation, the QP muscle was previously reported to have a close association with the AbH muscle at the level of the tarsal tunnel [4, 16]. These reports were not always clear and comparable, as they did not provide distinct definitions of the tunnel margins, nor other relations with any consistency. The current results therefore suggest that the QP muscle could also contribute to compression of neurovascular structures within the tarsal tunnel through hyperactivity, swelling or other deformations that consequently reduce the size of the tunnel.

Following dissection, the spatial relationships of the fibrous and muscular tissues within the tarsal tunnel were reassessed via the 3D model reconstructed in the present study. This approach was novel for the tarsal tunnel, as previous studies typically only rely on 2D images following dissection in Body Donor studies [4, 6, 10, 13], or illustrations [7]. In addition, clinical studies that have only utilised CT or MR imaging could not clearly identify individual soft tissues within the tarsal tunnel, or clearly identify the spatial relationships they have with one another [4].

There were a few limitations with the present study. A 2D reconstruction of the tarsal tunnel neurovasculature was not included in the Rhinoceros model, and the tendinous sheaths were incised to ensure the removal of the tendons which they encased. While the neurovasculature were not initially the focus of this study, future studies should consider reconstructing the tarsal tunnel as a single unit and model all its components. This may involve 3D Rhinoceros reconstructions alone or have the addition of CT and MR imaging prior to dissection and 3D modelling. This may improve the identification of structures of the tarsal tunnel in clinical CT and MR imaging, such as identifying which structures are compressing nerves to cause TTS.

The current results demonstrate that the nerves coursing through the tarsal tunnel could be compressed from various surrounding structures. These include the flexor retinaculum, the thick fibrous tissues separating it from the FDL tendon dorsally and/or the FHL tendon laterally, and the more medial fibrous and/or muscular tissues. These structures should therefore be considered during the treatment of TTS. Currently, only the flexor retinaculum is routinely considered and released [1–3], which may, in part, explain the poor clinical outcomes reported [3], including high revision rates [8]. The data presented here suggest that incision position, length and number of incisions may all need to be reconsidered when considering a surgical release for TTS.

## Conclusion

The findings from this study demonstrated that the tarsal tunnel is divided by a fibrous skeleton into at least five separate spaces. The neurovasculature also have a close association with the proximal AbH and QP muscle. The fibrous tissues may help determine, and contribute to, compartmentalisation of the foot, and, as a consequence of these relations, contribute to plantar compartment syndromes. These fibrous and muscular tissues may also contribute to the development of TTS and may have to be considered for its diagnosis and treatment.

## Data Availability

The data that support the findings of this study are not publicly available due to ethical restrictions. Access may be granted upon reasonable request via the corresponding author, subject to institutional approval and ethical compliance.
